# A simple spatial extension to the extended connectivity interaction features for binding affinity prediction

**DOI:** 10.1098/rsos.211745

**Published:** 2022-05-04

**Authors:** Oghenejokpeme I. Orhobor, Abbi Abdel Rehim, Hang Lou, Hao Ni, Ross D. King

**Affiliations:** ^1^ Department of Chemical Engineering and Biotechnology, University of Cambridge, Cambridge, UK; ^2^ Department of Biology and Biological Engineering, Chalmers University of Technology, Göteborg, Sweden; ^3^ Department of Mathematics, University College London, London, UK; ^4^ The Alan Turing Institute, London, UK

**Keywords:** machine learning, protein binding affinity prediction, scoring functions

## Abstract

The representation of the protein-ligand complexes used in building machine learning models play an important role in the accuracy of binding affinity prediction. The Extended Connectivity Interaction Features (ECIF) is one such representation. We report that (i) including the discretized distances between protein-ligand atom pairs in the ECIF scheme improves predictive accuracy, and (ii) in an evaluation using gradient boosted trees, we found that the resampling method used in selecting the best hyperparameters has a strong effect on predictive performance, especially for benchmarking purposes.

## 1. Background

It is commonplace to estimate the binding affinity of protein-ligand complexes using *in silico* scoring functions (models) built using statistical machine learning (ML) algorithms [1,2]. ML model performance on any predictive task largely depends on the quality of the descriptors used in building it. Therefore, one can argue that the performance of a scoring function is predicated on two key components: (i) the choice of ML algorithm and (ii) the quality of the input descriptors.

Among the many ML algorithms that have been used in constructing scoring functions [3–6], gradient boosted trees (GBTs) have been identified as one of the top performers [4,7,8]. Like most sophisticated ML algorithms, GBTs have several hyperparameters that require tuning to achieve the best performance on a given problem. The selection of the best hyperparameters usually involves a search through the hyperparameter space and their quality tested using some form of resampling [9]. While several search strategies for hyperparameter selection have been proposed [10], this is not the focus of this work. Here, we focus on the effect of resampling technique in the identification of optimal hyperparameters. We demonstrate empirically that the choice of resampling technique has strong effects on the choice of optimal hyperparameters, and by extension, the performance of a constructed scoring function. Although this is well known in the ML community, it is often a footnote or ignored in the protein-binding affinity prediction literature.

Several descriptors for the representation of protein-ligand complexes for use in building ML-based scoring functions have been proposed [8,11]. These descriptors often implement principles from extended connectivity fingerprints [12]. A recent approach which has been shown to achieve state-of-the-art performance is the extended connectivity interaction features [8] (ECIF), which identified 22 atom-types for proteins, and 70 atom-types for ligands based on connectivity. Pairs of these protein-ligand atom-types *within* a distance threshold are used as descriptors, where the value for each protein-ligand complex is the frequency of its occurrence. In this work, we extend this approach by distinguishing between shorter and longer descriptor interactions, and refer to this approach as pair distance ECIF (PDECIF). We evaluated the proposed approach using the comparative assessment of scoring functions (CASF) family of benchmark datasets [5,13,14], and demonstrate that PDECIF outperforms ECIF when paired with GBTs. Our contributions are as follows:

— We demonstrate the effect the choice of resampling technique when optimizing ML algorithm hyperparameters has on scoring function performance. Our analysis shows that the difference in predictive performance is not statistically significant. However, it is worth noting that the observed differences are indeed important, especially when comparing the performance of different scoring functions.
— We propose PDECIF, an extension to ECIF which outperforms its predecessor.

## 2. Methods

### 2.1. ECIF and PDECIF

A ligand descriptor in the ECIF framework consists of an atom’s symbol, explicit valence, number of attached heavy atoms, number of attached hydrogens, aromaticity and ring membership. Each of these properties can be represented textually where each property is separated by a semicolon. For example C;4;3;0;0;0. Protein descriptors are formulated in the same way where Protein Data Bank (PDB) residue and atom pairs map to an ECIF atom of the same form. For example, ASN-OXT maps to O;2;1;0;0;0. Given a protein-ligand complex and under a prespecified distance threshold, for example 6 Å, a valid ECIF descriptor consists of a protein-ligand atom pair of the form C;4;3;0;0;0-O;2;1;0;0;0. The assigned numerical value is the number of times it occurs in the complex under the distance threshold. In total, there are 1540 of such pairs, see the original work for the complete set [8]. In contrast to ECIF, rather than specify a maximum distance, we specify a distance below which we consider short and above which we consider long. The distance for long-range interactions is uncapped. So using the example for the ECIF descriptor above, we have the following descriptors for short and long, respectively; C;4;3;0;0;0-O;2;1;0;0;0-l and C;4;3;0;0;0-O;2;1;0;0;0-h. Note that the choice of ‘-l’ and ‘-h’ are simply as delineating symbols for the distances and have no intrinsic meaning.

### 2.2. Datasets

We performed our evaluation using the CASF 2007, 2013, 2016 and 2019 benchmark datasets [5,13,14]. They all have independent train and test sets, with 1090–210, 2764–195, 3772–285 and 9291–285 train–test samples for the aforementioned datasets, respectively. We used this existing split in our experiments. Note that the CASF 2019 set shares the same test set as the CASF 2016 set, albeit with more training samples. We read the raw data from PDBbind; where RDKit was incapable of reading the ligand files, Open Babel (v. 3.1.1) was used to convert these into mol files that were fully compatible with RDKit. All files were then saved in mol format before use. We considered four distance thresholds for both ECIF and PDECIF: 4 Å, 6 Å, 8 Å and 10 Å. Table 1 shows the number of features generated using ECIF and PDECIF at the different distance thresholds for the benchmark datasets. Note that the number of features for the ECIF datasets is never up to the reported 1540 in the original. This is because we only included features for which at least one complex has a non-zero value. We also considered a case were the ECIF and PDECIF datasets are augmented with 194 ligand features [15] generated using RDKit. The features are described in electronic supplementary material, S1.

**Table 1.** The number of features generated for each of the benchmark datasets using the ECIF and PDECIF approaches at different distances (angstroms).

benchmark	distance	ECIF	PDECIF
CASF 2007	4	856	2178
	6	1161	2482
	8	1244	2563
	10	1285	2595
CASF 2013	4	996	2290
	6	1226	2520
	8	1268	2561
	10	1288	2579
CASF 2016	4	1078	2485
	6	1332	2739
	8	1376	2781
	10	1399	2803
CASF 2019	4	1176	2584
	6	1362	2770
	8	1389	2795
	10	1402	2807

### 2.3. Evaluation set-up

We used GBT as our learner of choice. The hyperparameters were optimized using a grid search, and kept all but the number of rounds, max depth and learning rate as default values, where the number of rounds = (500, 1000, 1500, 2000), max depth = (2, 4, 6, 8) and learning rate = (0.001, 0.01, 0.1, 0.2, 0.3). Selection of the best combination of these parameters was performed using only the *training data* for each of the benchmarks. We considered three resampling approaches; train–test split (70–30%) and cross-validation (CV) with *k* = (5, 10). These were performed once and without repetition. Having identified the best performing hyperparameters and building the final model, we report the Pearson correlation coefficient (*R*) and root mean square error (RMSE). For both of the performance metrics, we report model performance on each benchmark’s test set. We performed feature selection on the overall best performing benchmark dataset and representation method pair using the Boruta algorithm [16] with a maximum of 500 runs, a *p*-value of 0.01, and a random forests [17] backend. The code used in generating the datasets and performing the experiments is available at https://github.com/oghenejokpeme/PDECIF. Links to all datasets used are also provided in the aforementioned repository.

## 3. Results and discussion

### 3.1. Comparison of ECIF and PDECIF

Our results show that PDECIF generally outperforms ECIF. This is irrespective of distance, the presence of ligand features or the resampling method used in tuning the predictive model. We also observed that the best performing representation for all the benchmark years included the ligand features, albeit at different distance thresholds. Crucially, we wanted to know if (i) the resampling method used in selecting the best set of hyperparameters for the predictive model affects performance, and (ii) there is a difference in predictive performance between the different representations. In the first case, paired *t*-tests indicate that the difference in performance is not statistically significant when the three resampling methods are paired up and compared across all benchmark years, distances and representations. However, with a significance level of 0.01 paired *t*-tests indicate a significant difference in performance when the different dataset representations are paired and compared across the different resampling methods for the latter (table 2).

**Table 2.** *P*-values from paired *t*-test statistical testing of the difference in predictive performance (*R*) between the considered representations across the different resampling methods.

representation pairs	train–test	CV5	CV10
ECIF − ECIF + Ligand	9.369 × 10^−5^	1.918 × 10^−4^	1.502 × 10^−4^
ECIF − PDECIF	2.133 × 10^−3^	6.220 × 10^−3^	6.419 × 10^−3^
ECIF − PDECIF + Ligand	5.471 × 10^−5^	1.271 × 10^−4^	1.391 × 10^−4^
ECIF + Ligand − PDECIF	5.364 × 10^−1^	1.388 × 10^−1^	1.188 × 10^−1^
ECIF + Ligand − PDECIF + Ligand	6.175 × 10^−3^	5.564 × 10^−4^	3.924 × 10^−3^
PDECIF − PDECIF + Ligand	3.688 × 10^−5^	6.243 × 10^−8^	1.052 × 10^−6^

On the CASF 2019 benchmark dataset, ECIF’s best performance (*R*/RMSE) is 0.842/1.258 and 0.858/1.208 without and with ligand features, respectively, both of which are at a distance of 10 Å. By contrast, PDECIF’s best performance is 0.854/1.235 and 0.862/1.204 without and with ligand features, respectively, where the former is at a distance of 4 Å and the latter is at a distance of 10 Å. It is worth noting that the results for ECIF differ from those reported by Sánchez-Cruz *et al.* [8], where ECIF’s best performance is 0.857/1.193 without ligand features, 0.866/1.169 with ligand features. In their work, the authors refer to the CASF-2019 benchmark dataset here as CASF-2016. Our results are more conservative. We believe this is the case because although the same raw files were retrieved from PDBbind, the preprocessing steps we used in generating the mol files which we then use in generating the representations differ. However, our results confirm the following findings reported in their prior work: (i) inclusion of ligand features significantly improves predictive performance, and (ii) ECIF and by extension PDECIF outperforms other state-of-the-art approaches such as convolutional neural network (CNN) architectures such as *K*_DEEP_ [3] (0.82/1.27) and TopBP-DL [18] (0.848/1.210). Furthermore, what we propose outperforms other state-of-the-art GBT-based scoring functions like AGL-Score [4] and EIC-Score [7] with Pearson *R* coefficients of 0.833 and 0.828 on the CASF-2016 benchmark. See tables 3 and 4 for our complete set of results.

**Table 3.** Predictive performance (*R*/RMSE) for the ECIF and PDECIF representations with and without the ligand features for the CASF 2007 and 2013 benchmark datasets when the hyperparameters for the predictive model are selected using the train–test and cross-validation (*k* = {5, 10}) resampling methods. For each benchmark year and distance pair, the best performing representation (with and without ligand features) and resampling method is in italics. The overall best performing combination for the given benchmark dataset is in boldface.

year—distance	representation	train–test	CV5	CV10
CASF 2007—4	ECIF	0.739/1.663	0.736/1.665	0.729/1.692
	PDECIF	*0.811/1.458*	0.807/1.468	0.802/1.482
	ECIF + ligand	0.759/1.583	0.787/1.562	0.783/1.562
	PDECIF + ligand	0.811/1.467	*0.817/1.468*	0.812/1.471
CASF 2007—6	ECIF	0.812/1.472	0.808/1.498	*0.821/1.450*
	PDECIF	0.803/1.494	0.808/1.467	0.806/1.472
	ECIF + ligand	0.814/1.459	0.820/1.455	0.812/1.460
	PDECIF + ligand	0.823/1.430	*0.826/1.428*	0.817/1.446
CASF 2007—8	ECIF	0.805/1.468	0.813/1.449	0.815/1.446
	PDECIF	*0.816/1.455*	0.812/1.450	0.811/1.460
	ECIF + ligand	0.815/1.472	0.818/1.443	0.820/1.442
	PDECIF + ligand	0.827/1.418	0.825/1.418	***0.828/1.408***
CASF 2007—10	ECIF	0.811/1.473	*0.820/1.429*	0.811/1.448
	PDECIF	0.811/1.476	0.808/1.476	0.807/1.481
	ECIF + ligand	0.802/1.496	0.820/1.461	0.817/1.438
	PDECIF + ligand	0.814/1.486	*0.822/1.444*	0.818/1.440
CASF 2013—4	ECIF	0.708/1.629	0.694/1.655	0.717/1.613
	PDECIF	0.762/1.522	0.773/1.499	*0.779/1.490*
	ECIF + ligand	0.777/1.484	0.776/1.480	0.778/1.481
	PDECIF + ligand	0.800/1.432	0.798/1.431	*0.801/1.429*
CASF 2013—6	ECIF	0.772/1.484	0.779/1.475	0.774/1.478
	PDECIF	*0.792/1.449*	0.786/1.461	0.783/1.467
	ECIF + ligand	0.801/1.419	0.791/1.437	0.790/1.439
	PDECIF + ligand	0.811/1.405	0.802/1.420	***0.817/1.384***
CASF 2013—8	ECIF	0.772/1.487	0.769/1.497	0.772/1.483
	PDECIF	0.774/1.485	0.784/1.459	*0.783/1.465*
	ECIF + ligand	0.799/1.420	0.797/1.423	0.799/1.422
	PDECIF + ligand	0.800/1.420	0.804/1.410	*0.806/1.402*
CASF 2013—10	ECIF	0.781/1.464	0.779/1.469	*0.786/1.458*
	PDECIF	0.780/1.469	0.775/1.478	0.778/1.472
	ECIF + ligand	0.800/1.420	0.798/1.416	*0.809/1.396*
	PDECIF + ligand	0.798/1.421	0.796/1.424	0.797/1.423

**Table 4.** Predictive performance (*R*/RMSE) for the ECIF and PDECIF representations with and without the ligand features for the CASF 2016 and 2019 benchmark datasets when the hyperparameters for the predictive model are selected using the train–test and cross-validation (*k* = {5, 10}) resampling methods. For each benchmark year and distance pair, the best performing representation (with and without ligand features) and resampling method is in italics. The overall best performing combination for the given benchmark dataset is in boldface.

year—distance	representation	train–test	CV5	CV10
CASF 2016—4	ECIF	0.752/1.497	0.752/1.495	0.748/1.501
	PDECIF	0.816/1.334	*0.823/1.317*	0.818/1.329
	ECIF + ligand	0.818/1.335	0.822/1.319	0.822/1.323
	PDECIF + ligand	*0.841/1.272*	0.840/1.273	0.839/1.275
CASF 2016—6	ECIF	0.808/1.343	0.811/1.335	0.802/1.353
	PDECIF	*0.833/1.277*	0.833/1.280	0.828/1.293
	ECIF + ligand	0.840/1.263	0.840/1.260	0.829/1.284
	PDECIF + ligand	0.843/1.252	0.840/1.258	*0.844/1.248*
CASF 2016—8	ECIF	0.806/1.343	0.804/1.350	0.797/1.361
	PDECIF	0.823/1.303	*0.829/1.290*	0.824/1.305
	ECIF + ligand	0.831/1.281	0.832/1.275	0.838/1.263
	PDECIF + ligand	0.831/1.276	*0.843/1.248*	0.842/1.256
CASF 2016—10	ECIF	0.815/1.320	0.812/1.328	0.816/1.314
	PDECIF	0.825/1.298	0.823/1.300	*0.830/1.288*
	ECIF + ligand	***0.844/1.245***	0.842/1.252	0.842/1.256
	PDECIF + ligand	0.842/1.252	0.844/1.246	0.839/1.260
CASF 2019—4	ECIF	0.793/1.424	0.795/1.417	0.791/1.426
	PDECIF	*0.854/1.235*	0.853/1.239	0.851/1.249
	ECIF + ligand	0.833/1.294	0.833/1.289	0.832/1.290
	PDECIF + ligand	0.859/1.217	0.855/1.223	*0.859/1.212*
CASF 2019—6	ECIF	0.832/1.284	0.837/1.272	0.833/1.284
	PDECIF	*0.850/1.236*	0.850/1.240	0.849/1.241
	ECIF + ligand	0.847/1.237	0.848/1.230	0.853/1.223
	PDECIF + ligand	0.859/1.208	*0.860/1.201*	0.860/1.204
CASF 2019—8	ECIF	0.831/1.290	0.836/1.281	0.839/1.268
	PDECIF	0.845/1.251	0.848/1.244	*0.849/1.239*
	ECIF + ligand	0.854/1.222	0.852/1.230	0.851/1.227
	PDECIF + ligand	0.857/1.215	***0.862/1.204***	0.854/1.222
CASF 2019—10	ECIF	0.832/1.282	0.842/1.258	0.837/1.271
	PDECIF	0.848/1.245	0.849/1.248	*0.851/1.236*
	ECIF + ligand	0.856/1.211	0.858/1.208	0.854/1.217
	PDECIF + ligand	0.855/1.215	*0.859/1.207*	0.857/1.203

### 3.2. Feature importance

We performed feature selection on the CASF 2019 benchmark training dataset with the PDECIF representation at a distance of 8 Å augmented with the ligand features, as it was the best performing (table 4). This dataset has 2912 features. The Boruta feature selection algorithm identified 817 confirmed important features. The top 20 of these features by mean importance in descending order are: C;4;3;1;0;0-C;4;3;0;1;1-h, N;3;2;1;0;0-C;4;3;0;1;1-h, O;2;1;0;0;0-C;4;3;0;1;1-h, C;4;3;0;0;0-C;4;3;0;1;1-h, Crippen_MolLogP, C;4;1;3;0;0-C;4;3;0;1;1-h, C;4;2;1;1;1-C;4;3;0;1;1-h, C;4;3;0;1;1-C;4;3;0;1;1-h, SlogP_VSA2, Chi2v, C;4;2;2;0;0-C;4;3;0;1;1-h, O;2;1;0;0;0-C;4;3;0;1;1-l, Chi3v, C;4;3;0;1;1-C;4;2;1;1;1-h, C;4;1;3;0;0-C;4;3;0;1;1-l, C;4;2;1;1;1-C;4;2;1;1;1-l, Chi1v, MaxAbsPartialCharge, Chi4v, C;4;2;1;1;1-C;4;3;0;1;1-l. The full set of important features are provided in the electronic supplementary material.

It is worth noting that 121 of the 194 ligand features we considered were selected as part of the 817 important features. Having identified these features using the training set, we performed additional experiments using just them and the same tuning configuration discussed in the previous section. The performance (*R*/RMSE) for the train–test, CV5 and CV10 resampling methods are 0.852/1.224, 0.851/1.224 and 0.849/1.228 respectively. This means that they achieve approximately 99.4%, 98.7% and 99.4% of the full dataset performance (see row ‘CASF 2019—8’ and entry ‘PDECIF + ligand’ in table 4).

## 4. Conclusion

In this paper, we have presented a simple extension to the ECIF representation approach for the *in silico* prediction of protein binding affinity. Our results show that the extension significantly outperforms the base approach on the CASF benchmark datasets. Furthermore, we show that for GBTs, the resampling method used in optimizing the hyperparameters affects predictive accuracy. This is particularly important when comparing against other scoring functions, where progress is measured using performance on benchmark datasets. However, our experiments show that the difference in performance gain is not statistically significant.

## Data Availability

Data and relevant code for this research work are stored in GitHub: https://github.com/oghenejokpeme/PDECIF and have been archived within the Zenodo repository: https://doi.org/10.5281/zenodo.6448428.
